# Long-term lifestyle effects of prehabilitation in colorectal cancer

**DOI:** 10.2340/1651-226X.2026.45608

**Published:** 2026-05-27

**Authors:** Lisa D. Geomini, Barend J. Spanninga, Margot H. M. Heijmans, Gerrit D. Slooter

**Affiliations:** Department of Surgery, Máxima MC, Veldhoven, The Netherlands

**Keywords:** Colorectal cancer, prehabilitation, lifestyle, patient perspective

## Abstract

**Background and purpose:**

Prehabilitation—a multimodal intervention aimed at optimizing patients’ physical, nutritional and psychological status prior to surgery—has been shown to improve postoperative outcomes. However, the long-term impact of such programs on lifestyle remains poorly understood. Aim is to evaluate long-term lifestyle changes following a 4-week prehabilitation program prior to elective colorectal cancer (CRC) surgery and to compare patient-reported outcomes over a 24-month postoperative period between prehabilitation and control groups.

**Patient/material and methods:**

This single-center cross-sectional study combined a prehabilitation specific questionnaire with longitudinal data from the Prospective Dutch Colorectal Cancer (PLCRC) cohort. Questionnaires were distributed to CRC patients who underwent prehabilitation between 2021 and 2023. Longitudinal data—including validated measures of physical activity and quality of life—were compared between prehabilitation and control groups at 6, 12, and 24 months post-surgery.

**Results:**

A total of 137 prehabilitation questionnaires were completed. Over half of the patients reported sustained lifestyle changes, including increased physical activity, healthier dietary habits and smoking cessation. However, longitudinal analyses of the PLCRC cohort (n=140) showed no significant differences in physical activity or quality of life between the prehabilitation and control groups.

**Interpretation:**

Patients reported perceived meaningful, sustained, long-term lifestyle improvements following prehabilitation. The positive patient perspective may help support the broader implementation of prehabilitation in CRC care and highlights its potential to facilitate long-term lifestyle changes. However, these improvements were not reflected in the PLCRC data, likely due to high variability and the limited sample size of the dataset.

## Graphical abstract

**Figure:**
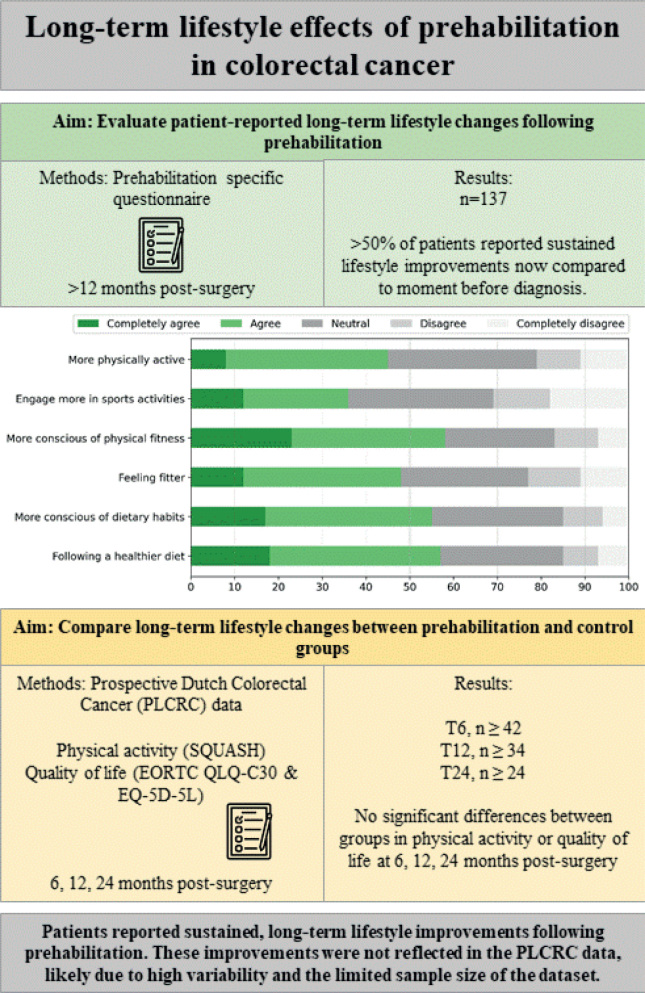

## Introduction

Prehabilitation, which aims to optimize patients’ fitness prior to surgery, has been shown to improve preoperative functional capacity and reduce postoperative complications, thereby decreasing morbidity and mortality rates [1–7]. This multifaceted approach includes various preoperative interventions such as (supervised) physical training, nutritional assessment and intervention, cessation of smoking and alcohol consumption, patient blood management (including correction of preoperative anemia and hyperglycemia), and psychological support when indicated [2–6, 8, 9]. Patients get the chance to improve their fitness, their nutritional habits and lifestyle. Accordingly, prehabilitation patients improved their steep ramp test and their muscle strength postoperatively [10]. Prehabilitation also positively impacts hospital resource utilization and reduces healthcare costs by decreasing complications, length of hospital stay, intensive care use, and readmissions [11]. As a result, prehabilitation has been implemented for patients undergoing especially colorectal cancer (CRC) surgery in multiple hospitals across the Netherlands [8].

Successful implementation is contingent upon patient adherence to the program. While previous, small-scale qualitative interviews [12] and proof-of-concept studies [13] have explored this topic, patient perceptions with the currently implemented program in a larger cohort remain unknown.

After participation in a prehabilitation program, patients often report adopting healthier lifestyle behaviors during follow-up. In this context, lifestyle refers to modifiable health‑related behaviors such as physical activity, dietary habits, smoking behavior and psychosocial coping, all of which are increasingly recognized as important across the cancer care continuum. If prehabilitation supports sustained adoption of these behaviors, its benefits may extend beyond the perioperative period, potentially improving quality of life and broader societal outcomes [14].

However, evidence regarding the long-term lifestyle effects of prehabilitation remains limited and inconsistent. Previous studies have predominantly focused on short-term outcomes or lacked longitudinal follow-up [15, 16], making it difficult to draw conclusions about sustained behavioral change. Moreover, maintaining lifestyle changes over time is inherently challenging, with a well-documented gap between initial behavioral intention and sustained adherence, often accompanied by behavioral relapse [17]. Consequently, the extent to which prehabilitation leads to enduring lifestyle change has not yet been systematically evaluated.

The aim of the present manuscript was twofold. First, we aimed to explore patients’ experiences and self‑perceived long‑term lifestyle effects of prehabilitation over a 12‑month follow‑up period using a prehabilitation‑specific questionnaire. Second, to place these patient‑reported perceptions in a comparative context, we aimed to assess long‑term patient‑reported outcomes at 24 months by comparing patients who did and did not undergo prehabilitation using longitudinal prospective data.

## Patients/material and methods

### Study design

This mixed-design study consisted of a single-center cross-sectional questionnaire study and a longitudinal cohort study using prospectively collected data from the Prospective Dutch Colorectal Cancer (PLCRC) cohort, a nationwide multidisciplinary observational cohort in the Netherlands. All patients with CRC, small bowel adenocarcinoma, and anal cancer were eligible for inclusion. After patients have given their informed consent, longitudinal clinical data were registered, and patient reported outcomes (PROMs) were collected [18]. Approval from the medical ethical committee (METC) was requested; however, formal approval was deemed unnecessary under Dutch law (METC, Máxima MC). The study was approved by the Institutional Review Board of Máxima MC (2024.0096). The study design comprised two components. First, a cross-sectional prehabilitation questionnaire assessed persistent effects of prehabilitation on daily habits, lifestyle and experiences with the prehabilitation program. Second, relevant PROMs from the longitudinal PLCRC cohort were analyzed to be able to compare long-term effects between patients who participated in the prehabilitation program and patients who did not. Because the prehabilitation questionnaire cohort and the PLCRC cohort were not identical and could partially overlap, analyses were performed separately for each study component.

### Prehabilitation program

At Máxima MC, prehabilitation is implemented as a multimodal standard-of-care program for patients with CRC undergoing elective surgery, in accordance with the national prehabilitation protocol [19]. The program has a duration of 4 weeks and includes supervised physiotherapy sessions three times per week, each lasting 1 hour. In addition, patients receive nutritional counseling by a dietitian, including daily protein and vitamin supplementation. Psychological support is provided by a dedicated case manager. When indicated, additional interventions such as optimization of anemia status and a smoking cessation program are incorporated.

### Prehabilitation questionnaire

The prehabilitation questionnaire was sent to all eligible patients (≥18 years at time of surgery) who participated in the prehabilitation program prior to elective CRC surgery between January 2021 and September 2023 at Máxima MC and were alive at the time of questionnaire distribution (September 2024). Only patients who provided consent and completed the questionnaire adequately were included. Baseline characteristics—age at surgery, sex, body mass index (BMI), smoking habits (yes/no), and American Society of Anesthesiologists (ASA) score—were extracted from electronic medical records.

Patients received the questionnaire by e-mail, with a reminder sent 1 week later if applicable. Non-responders were subsequently sent a paper version by mail. Questionnaires were included for analysis if at least one question related to the prehabilitation program was answered. The questionnaire primarily invited patients to rate their current lifestyle behavior such as physical activity, dietary habits and smoking status in comparison with their habits prior to diagnosis. Participants responded to a series of positively framed statements (e.g., “I am more physically active than before my diagnosis”) using a five-point Likert scale ranging from “completely agree” to “completely disagree.” In addition, the questionnaire assessed participants’ experiences with the prehabilitation program.

### PLCRC PROM questionnaires

PROMs from the PLCRC cohort were requested for all patients who underwent elective CRC surgery at Máxima MC and participated in the PLCRC study, which has been extensively described elsewhere [18]. The PLCRC cohort comprised participants between October 2018 (initiation of PLCRC) and November 2024, and analyses were based on questionnaires collected during this inclusion period. Since the PLCRC cohort was initiated at Máxima MC before prehabilitation implementation (January 2021), patients were divided into two groups: those who received prehabilitation (prehab group) and those who did not (control group). Participants were included only if they had completed the initial questionnaires within 30 days before or after surgery, and at least 6 months prior to the date of data collection. Baseline characteristics were obtained from PLCRC data and electronic medical records, including age at surgery, sex, BMI, smoking habits (yes/no), and surgical procedure.

PROM data comprised of a comprehensive set of questionnaires administered at baseline (T0, time of inclusion in the PLCRC cohort) and follow-up at 3, 6, 12, 18, 24, 36, 48, and 60 months post-inclusion. Given the interest in long-term outcomes, yet taking into account the declining response rates over time, analyses were limited to data collected at 6, 12, and 24 months. It should be noted that T0 corresponded to the moment of PLCRC inclusion, which did not necessarily coincide with the time of diagnosis. As a result, T0 could correspond to the start of the prehabilitation program, a point midway through the program, or just prior to surgery. Data at T0 were therefore excluded from between-group comparisons, due to the considerable influence of the prehabilitation program on activity levels and quality of life at this time point. During follow-up, PROM assessments administered specifically in the context of metastatic disease were excluded, as these reflect a distinct clinical phase with determinants of quality of life and functioning that are not comparable to those of non-metastasized patients.

PROM questionnaire scores were calculated according to their respective scoring manuals.

Physical activity was evaluated using the Short QUestionnaire to ASsess Health-enhancing physical activity (SQUASH), which measures frequency, duration, and intensity of activities over 1 week. Metabolic Equivalent of Task (MET) units were calculated for each activity to allow objective comparisons across different types of physical activity. One MET-hour corresponds to the energy expenditure of sitting quietly for 1 hour, while jogging at a self-selected pace corresponds to approximately 7 MET-hours per hour [20]. In addition to MET-hours, an activity score was computed for each activity, incorporating the MET value, the participant’s age, and the self-reported intensity. Both MET-hours per week and activity scores were calculated for sports activities, general daily activities, and total physical activity, resulting in six distinct outcome variables. To ensure data validity, responses were screened for implausible values (e.g., reporting more hours of cycling per week than feasible). Outliers, defined as values exceeding three times the interquartile range (IQR), were manually reviewed. Participants were excluded from analyses at the relevant time point if their responses, although technically possible, were deemed highly unrealistic—for example, reporting gardening 24 hours per day, 7 days per week.

Health-related quality of life was measured using the EQ-5D-5L instrument [21]. The EQ-5D-5L index value (EQ-index), ranging from -0.446 to 1.00, was derived using the Dutch value set, while the visual analog scale (EQ-VAS), ranging from 0 to 100, for health status was analyzed separately [22]. Quality of life was also assessed with the EORTC QLQ-C30 questionnaire. Scores for functioning scales (physical, role, emotional, cognitive, and social functioning) and global health status were computed on a 0–100 scale, with lower scores indicating poorer functioning [23]. Additionally, an EORTC QLQ-C30 summary score was calculated [24].

Missing data were handled according to the guidelines specified in each questionnaire’s manual [20, 23]. Analyses were performed using available data only, and no imputation was applied.

### Statistical analysis

Baseline characteristics were compared between participants and non-participants of the prehabilitation questionnaire to assess potential non-response bias. Prehabilitation questionnaire results were reported descriptively. For the PROM questionnaire data, baseline characteristics were compared between the prehab and control groups. Two-sided analyses were conducted using IBM SPSS Statistics (version 29.0, Armonk, NY, United States). Continuous variables are presented as mean ± standard deviation (SD) or median with interquartile range (IQR), depending on data distribution, and were analyzed using independent *t* tests or Mann–Whitney *U* tests. Categorical variables are presented as counts and percentages and analyzed using the Chi-square, Fishers’ exact, or Freeman-Halton tests as appropriate. Statistical significance was set at *p* < 0.05.

Comparison of PROM questionnaire scores between the prehab and control groups at each time point was performed using independent *t* tests or Mann–Whitney *U* tests. Statistical significance was set at *p* < 0.05, and to account for multiple comparisons, the Holm–Bonferroni method was used.

## Results

The results of the prehabilitation questionnaire and the PLCRC PROM questionnaire are presented separately below. Of note, 54 patients were included in both sections, but were analyzed separately according to the respective study designs.

### Prehabilitation questionnaire—population

**Figure 1:**
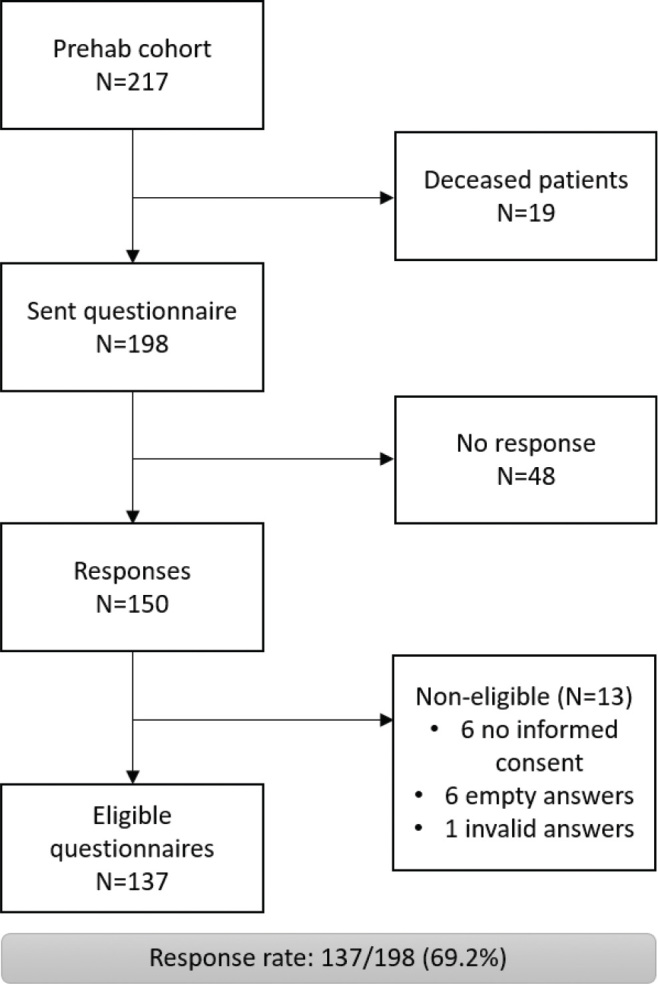
Prehabilitation questionnaire—flowchart for eligible questionnaires of the prehabilitation cohort (January 2021–September 2023).

Between January 2021 and September 2023, a total of 217 patients participated in the prehabilitation program for elective CRC surgery in Máxima MC. Of these, 198 patients were alive and eligible to receive the questionnaire in September 2024. In total, 137 (69%) questionnaires were eligible for analysis, and the flowchart is depicted in Figure 1. Patient characteristics of participants and non-participants are shown in Table 1. Groups were comparable regarding sex, BMI, ASA score and smoking status. Participants were significantly younger than non-participants (*p* < 0.001).

**Table 1:** Baseline characteristics—prehabilitation questionnaire.

Characteristics	Participants *n* = 137	Non-participants *n* = 61	*P*
Age, years, median (IQR)	69.0 (62.0–75.5)	77.0 (67.5–81.0)	**< 0.001**
Sex, male, *n* (%)	75 (54.7)	28 (45.9)	0.250
BMI, kg/m^2^, median (IQR)	25.7 (23.3–28.7)	25.1 (23.0–28.3)	0.737
ASA score, *n* (%)			0.366
I	5 (3.6)	4 (6.6)	
II	92 (67.2)	35 (57.4)	
III	38 (27.7)	22 (36.1)	
IV	2 (1.5)	0 (0.0)	
Active smoker*, *n* (%)	14 (10.2)	8 (13.1)	0.549

BMI: Body Mass Index; ASA: American Society of Anesthesiologists; IQR: interquartile range. Significance was set at *p* < 0.05.

* Please note the discrepancy between the number of active smokers documented in this Table (*n* = 14, medical records) and the self-reported number of smokers (*n* = 17), which may be attributed to the nature of self-reported information.

### Prehabilitation questionnaire—outcomes

**Figure 2:**
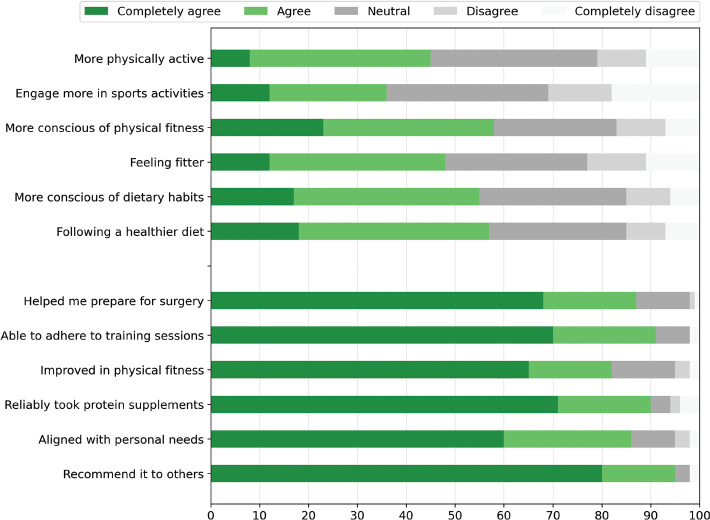
Long-term lifestyle effects and patient experience of prehabilitation. Patients were asked to indicate the level of agreement with six statements comparing their current lifestyle habits to those prior to symptom onset or diagnosis and six statements regarding the prehabilitation program.

The first section of the questionnaire assessed how participants’ current lifestyle habits compared to those prior to diagnosis or symptom onset (Figure 2). For all subquestions, approximately 50% of participants agreed or completely agreed with statements suggesting a positive long-term effect of prehabilitation. Detailed scores for the individual subquestions are presented in Figure 2.

The second section of the questionnaire assessed patients’ experiences with the prehabilitation program (Figure 2). The program received a median overall rating of 9 (IQR 8–10). Moreover, for all items in this section, more than 80% of participants either agreed or completely agreed with the statements. This high level of agreement indicates that patients had a generally positive experience with the program. Detailed scores for the individual sub-questions are presented in Figure 2.

Finally, 17 patients reported being active smokers at the time of their diagnosis. Among these individuals, eight have completely quit smoking, four report a reduction in smoking, while the remaining five indicate smoking at the same or increased levels compared to the time of diagnosis. Notably, 50% of the participants who reported smoking less, attributed their behavioral change to the support provided by the prehabilitation program.

### PLCRC PROM questionnaires—population

**Figure 3:**
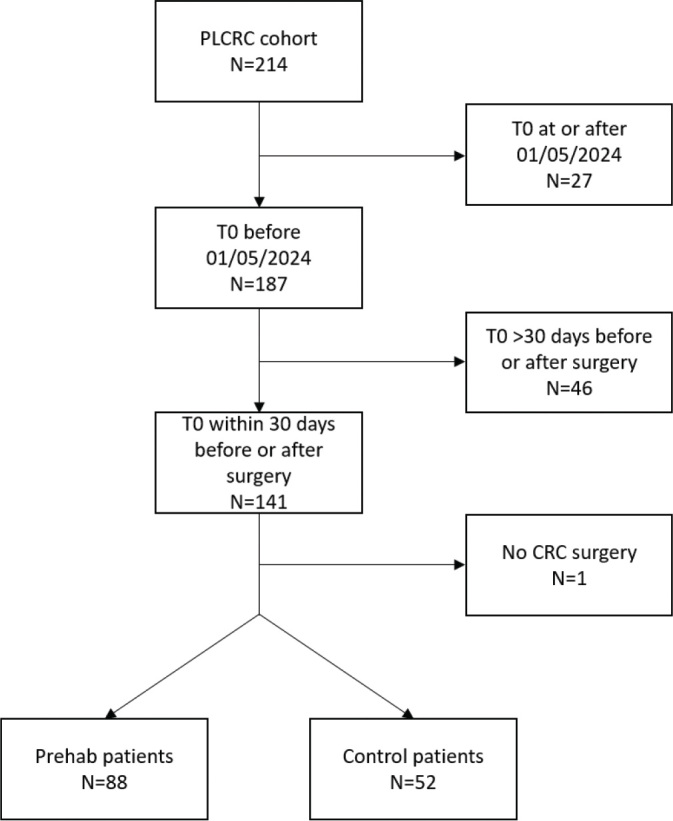
PLCRC PROM questionnaire—flowchart for eligible prehabilitation and control patients of the PLCRC cohort (October 2018–November 2024). PLCRC: Prospective Dutch Colorectal Cancer; PROM: patient reported outcomes; CRC: colorectal cancer.

The PLCRC dataset of Máxima MC included 214 patients, of whom 116 underwent prehabilitation and 98 did not. A total of 140 participants were eligible for analysis, comprising 88 in the prehab group and 52 in the control group (Figure 3). The minimal number of participants completing the questionnaires declined over time. By 24 months, the number of prehab and control group respondents was 27 and 24, respectively.

The T0 questionnaires were completed between April 2019 and April 2024. Baseline characteristics are presented in Table 2, demonstrating comparability between the two groups.

**Table 2:** Baseline characteristics—PLCRC PROM questionnaires.

Characteristics	Prehab *n* = 88	Control *n* = 52	*P*
Age, years, mean (SD)	68.0 (11.1)	67.3 (9.6)	0.702
Sex, male, *n* (%)	50 (56.8)	30 (57.7)	0.920
BMI, kg/m^2^, mean (SD)	26.1 (4.0)	27.1 (4.7)	0.200
ASA score, *n* (%)			0.328
I	4 (4.5)	5 (9.4)	
II	62 (70.5)	31 (60.4)	
III	21 (23.9)	14 (26.4)	
IV	1 (1.1)	2 (3.8)	
Active smoker, *n* (%)	4 (4.5)	3 (5.8)	0.711
Procedure, *n* (%)			0.288
Right sided hemicolectomy	38 (43.2)	24 (46.2)	
Sigmoid	16 (18.2)	11 (21.2)	
TME/PME	12 (13.6)	8 (15.4)	
Left sided hemicolectomy	8 (9.1)	3 (5.8)	
APR	5 (5.7)	5 (9.6)	
LAR	7 (8.0)	0 (0.0)	
Transverse colectomy	2 (2.3)	0 (0.0)	
Subtotal colectomy	0 (0.0)	1 (1.9)	

BMI: Body Mass Index; ASA: American Society of Anesthesiologists; TME: Total Mesorectal Excision; PME: Partial Mesorectal Excision; APR: Abdominoperineal Resection; LAR: Low Anterior Resection; SD: standard deviation. Significance was set at *p* < 0.05.

### PLCRC PROM questionnaires—outcomes

**Figure 4:**
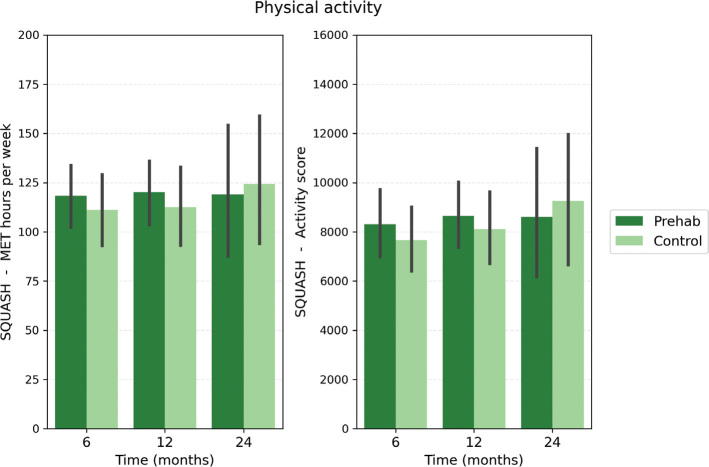
Total physical activity outcomes (MET hours per week and activity score) per time point per group. Bar plots representing mean and standard error. MET: Metabolic Equivalent of Task.

Physical activity questionnaire scores at time points T6, T12, and T24 for both groups are presented in Figure 4. No differences were observed between the prehab and control groups for any of the SQUASH subscale scores (all *p* > 0.05, Figure 4, Supplementary Table 1). Because none of the unadjusted *p*-values approached statistical significance, applying a Holm–Bonferroni correction for these comparisons was deemed unnecessary.

**Figure 5:**
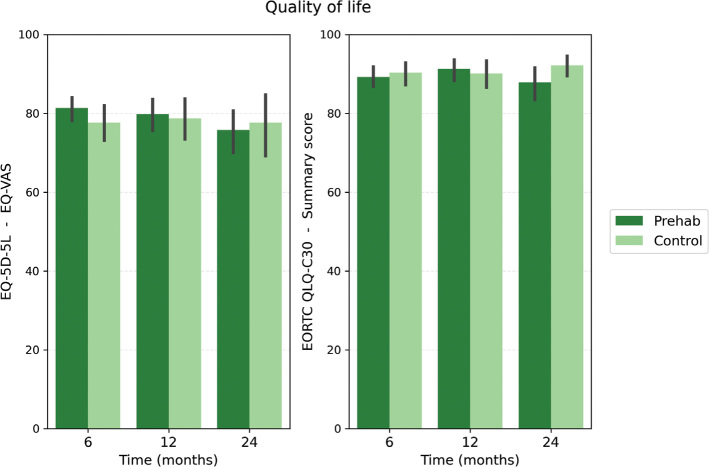
Quality of life outcomes (EQ-5D-5L EQ-VAS score and EORTC QLQ-C30- summary score) per time point per group. Bar plots representing mean and standard error. VAS: visual analog scale.

Quality of life questionnaire scores at time points T6, T12, and T24 for both groups are shown in Figure 5. To account for multiple testing, the Holm–Bonferroni correction was applied across the 27 group comparisons. None of the tests remained statistically significant after correction (all adjusted *p*‑values = 1.00). Consequently, no differences were detected between the prehab and control groups in EQ-5D-5L or EORTC QLQ-C30 subscale scores (Figure 5, Supplementary Table 2).

## Discussion and conclusion

This explorative study investigated the long-term lifestyle effects of a prehabilitation program preceding elective CRC surgery and patients’ experience with the program. Participants reported sustained improvements in daily physical activity, greater awareness of their physical fitness and healthier dietary habits compared with their pre-diagnosis situation. These positive trends were not reflected in the PLCRC data, likely due to substantial variability and the limited sample size. Overall, patient satisfaction and adherence to the prehabilitation program were high; participants indicated that the program met their needs and that they would recommend it to others.

Literature on long-term effects of prehabilitation on lifestyle habits is sparse and focuses on (disease free) survival or hospital admission [25, 26]. The present study is, to our knowledge, the first evaluation of long-term effects over a period of 24 months of prehabilitation on physical activity, quality of life, and daily functioning. Previous work demonstrates that adherence to healthy lifestyle recommendations—encompassing diet, physical activity and body composition—is positively associated with health‑related quality of life and functional outcomes among long‑term CRC survivors [27]. Although this crosssectional study does not examine prehabilitation nor assess longitudinal changes attributable to a structured preoperative intervention, its results underscore the broader relevance of sustained healthy lifestyle behaviors for long‑term survivorship outcomes. Consequently, the study indirectly reinforces the rationale for investigating whether prehabilitation can induce durable improvements in lifestyle, physical activity and quality of life, as addressed in our 24‑month follow‑up evaluation.

Approximately 50% of participants agreed or completely agreed with statements suggesting a positive long-term effect of prehabilitation. Ferreira et al. [28] previously reported that 94% of patients intended to continue exercising after surgery. This intention was assessed through a qualitative descriptive study in which 52 cancer patients completed a structured questionnaire evaluating their prehabilitation experience and their motivation to maintain exercise habits [28]. We observed that 45% of participants reported being more physically active now compared to the period before their diagnosis. Due to the phrasing of the question, it is not possible to determine whether the remaining 55% are currently equally or less physically active. Taken together, these findings indicate that our results align closely with previous evidence, and the fact that at least 45% of participants are certainly more active—with the remaining group potentially equally active—suggests a remarkably favorable and comparable long‑term impact of prehabilitation on physical activity levels. Potential differences between individuals’ motivation to sustain lifestyle changes and the extent to which such behaviors are ultimately carried out are well‑recognized in the literature, as motivation often exceeds actual long‑term implementation [29, 30].

Previous research into patient experience of prehabilitation programs focuses on facilitators and barriers to increase compliance of the program [12, 28, 31–33]. A general theme in these articles is the importance of a patient-centered and personalized program combined with education on the benefits of prehabilitation before surgery. 86% of patients stated the program met their personal needs, with over 90% reporting high compliance to the program, which is in line with the literature [4, 13, 28, 32]. The importance of adequate support and guidance in the period prior to surgery has been shown before [12, 34]. In accordance, 87% of patients experienced that the program helped them prepare for surgery.

Although positive long-term outcomes of prehabilitation were suggested by self-reported responses in the prehabilitation questionnaire, these findings should be interpreted with caution. The reported long‑term lifestyle improvements reflect self‑perceived changes rather than objectively measured differences. Beyond previously reported short-term cost reductions [11], sustained improvements in health, functioning or quality of life may hypothetically translate into reduced long-term societal costs, such as decreased work absenteeism or healthcare utilization. However, examining such societal costs is complex and influenced by numerous contextual factors, and was therefore beyond the scope of the present study.

Several limitations should be considered when interpreting these findings. The prehabilitation questionnaire was not formally validated and consisted primarily of positively framed questions, which may have introduced response bias. Furthermore, outcomes were based on self‑reported data, reflecting perceived rather than objectively measured long‑term lifestyle changes and may therefore be subject to recall bias. Survivorship bias and responder bias are also likely present, as only patients who remained alive and responded during follow‑up were included and questionnaire participants tended to be younger than non‑participants. The relatively small sample size and loss to follow-up in the PLCRC PROM data limited statistical power over the 24‑month follow‑up, increasing the likelihood that true between‑group differences may have gone undetected. In addition, residual confounding cannot be excluded due to the non‑randomized study design. Lastly, the lack of a genuine baseline measurement implies that comparisons at the follow‑up time points should be interpreted as cross‑sectional snapshots rather than true longitudinal trends. Taken together, these limitations underscore the exploratory nature of the study and highlight the need for future research using larger cohorts, validated (pre-intervention) instruments and objective outcome measures.

At least 1 year after completing prehabilitation, patients reported clear and meaningful long‑term benefits, including greater awareness of their lifestyle habits and increased daily physical activity compared to the period before diagnosis. These self‑reported improvements suggest that prehabilitation may support sustained lifestyle changes beyond the immediate perioperative phase, even though such effects were not observable in the PLCRC dataset—likely due to substantial variability and the limited sample size of the available data. Complementing these long‑term patient‑reported outcomes, the study also demonstrated high levels of patient satisfaction and patients mainly reported strong compliance with the multimodal prehabilitation program prior to elective CRC surgery. The consistently positive patient experience may therefore act as an important facilitator for broader implementation of prehabilitation within routine clinical practice.

## Supplementary Material



## Data Availability

Upon reasonable request.

## References

[1] Molenaar CJL, Minnella EM, Coca-Martinez M, Ten Cate DWG, Regis M, Awasthi R, et al. Effect of multimodal prehabilitation on reducing postoperative complications and enhancing functional capacity following colorectal cancer surgery: the PREHAB randomized clinical trial. JAMA Surg. 2023;158(6):572–81. 10.1001/jamasurg.2023.0198 36988937 PMC10061316

[2] Barberan-Garcia A, Ubre M, Roca J, Lacy AM, Burgos F, Risco R, et al. Personalised prehabilitation in high-risk patients undergoing elective major abdominal surgery: a randomized blinded controlled trial. Ann Surg. 2018;267(1):50–6. 10.1097/SLA.0000000000002293 28489682

[3] Hughes MJ, Hackney RJ, Lamb PJ, Wigmore SJ, Christopher Deans DA, Skipworth RJE. Prehabilitation before major abdominal surgery: a systematic review and meta-analysis. World J Surg. 2019;43(7):1661–8. 10.1007/s00268-019-04950-y 30788536

[4] Minnella EM, Bousquet-Dion G, Awasthi R, Scheede-Bergdahl C, Carli F. Multimodal prehabilitation improves functional capacity before and after colorectal surgery for cancer: a five-year research experience. Acta Oncol. 2017;56(2):295–300. 10.1080/0284186X.2016.1268268 28079430

[5] van Rooijen SJ, Molenaar CJL, Schep G, van Lieshout R, Beijer S, Dubbers R, et al. Making patients fit for surgery: introducing a four pillar multimodal prehabilitation program in colorectal cancer. Am J Phys Med Rehabil. 2019;98(10):888–96. 10.1097/PHM.0000000000001221 31090551

[6] West MA, Wischmeyer PE, Grocott MPW. Prehabilitation and nutritional support to improve perioperative outcomes. Curr Anesthesiol Rep. 2017;7(4):340–9. 10.1007/s40140-017-0245-2 29200973 PMC5696441

[7] Levett DZ, Edwards M, Grocott M, Mythen M. Preparing the patient for surgery to improve outcomes. Best Pract Res Clin Anaesthesiol. 2016;30(2):145–57. 10.1016/j.bpa.2016.04.002 27396803

[8] Molenaar CJL, Reudink M, Sabajo CR, Janssen L, Roumen RMH, Klaase JM, et al. Prehabilitation for patients with colorectal cancer: a snapshot of current daily practice in Dutch hospitals. Perioper Med (Lond). 2023;12(1):15. 10.1186/s13741-023-00299-y 37158927 PMC10165784

[9] van Rooijen S, Carli F, Dalton SO, Johansen C, Dieleman J, Roumen R, et al. Preoperative modifiable risk factors in colorectal surgery: an observational cohort study identifying the possible value of prehabilitation. Acta Oncol. 2017;56(2):329–34. 10.1080/0284186X.2016.1267872 28067102

[10] Ten Cate DWG, Sabajo CR, Molenaar CJL, Janssen L, Bongers BC, Slooter GD. Multimodal prehabilitation in elective oncological colorectal surgery enhances preoperative physical fitness: a single center prospective real-world data analysis. Acta Oncol. 2024;63:35–43. 10.2340/1651-226X.2024.20287 38477370 PMC11332481

[11] Sabajo CR, Ten Cate DWG, Heijmans MHM, Koot CTG, van Leeuwen LVL, Slooter GD. Prehabilitation in colorectal cancer surgery improves outcome and reduces hospital costs. Eur J Surg Oncol. 2024;50(1):107302. 10.1016/j.ejso.2023.107302 38043359

[12] Sier MAT, Cox M, Tweed T, Servaas N, Greve JWM, Stoot J. Participation and compliance in a multimodal prehabilitation program for colorectal cancer (PACE): a qualitative study. Patient Prefer Adherence. 2024;18:2709–20. 10.2147/PPA.S481567 39759885 PMC11697675

[13] Tweed TTT, Sier MAT, Van Bodegraven AA, Van Nie NC, Sipers W, Boerma EG, et al. Feasibility and efficiency of the BEFORE (Better Exercise and Food, Better Recovery) prehabilitation program. Nutrients. 2021;13(10):3493. 10.3390/nu13103493 34684494 PMC8538645

[14] Fong M, Kaner E, Rowland M, Graham HE, McEvoy L, Hallsworth K, et al. The effect of preoperative behaviour change interventions on pre- and post-surgery health behaviours, health outcomes, and health inequalities in adults: a systematic review and meta-analyses. PLoS One. 2023;18(7):e0286757. 10.1371/journal.pone.0286757 37406002 PMC10321619

[15] Molenaar CJ, van Rooijen SJ, Fokkenrood HJ, Roumen RM, Janssen L, Slooter GD. Prehabilitation versus no prehabilitation to improve functional capacity, reduce postoperative complications and improve quality of life in colorectal cancer surgery. Cochrane Database Syst Rev. 2023;5(5):CD013259. 10.1002/14651858.CD013259.pub3 37162250 PMC10171468

[16] Suarez-Alcazar MP, Folch Ayora A, Muriach M, Recacha-Ponce P, Garcia-Roca ME, Coret-Franco A, et al. Multimodal prehabilitation in colorectal cancer: improving fitness, lifestyle, and post-surgery outcomes. Healthcare (Basel). 2025;13(9):1083. 10.3390/healthcare13091083 40361861 PMC12071798

[17] Jekauc D, Voelkle MC, Sniehotta FF, Nigg CR. Beyond the cross-section: rethinking the intention-behaviour gap through a conceptual and methodological lens. Br J Health Psychol. 2026;31(1):e70046. 10.1111/bjhp.70046 41467472 PMC12750492

[18] Burbach JP, Kurk SA, Coebergh van den Braak RR, Dik VK, May AM, Meijer GA, et al. Prospective Dutch colorectal cancer cohort: an infrastructure for long-term observational, prognostic, predictive and (randomized) intervention research. Acta Oncol. 2016;55(11):1273–80. 10.1080/0284186X.2016.1189094 27560599

[19] Nederlandse Vereniging voor Heelkunde. Standpunt prehabilitatie: voorbereiding van patiënten met colorectaal carcinoom op de operatie. Utrecht: Nederlandse Vereniging voor Heelkunde; 2023.

[20] Wendel-Vos G, Schuit AJ. Handleiding SQUASH. Bilthoven: Centrum voor preventie en zorgonderzoek, Rijksinstituut voor Volksgezondheid en milieu. 2004.

[21] Herdman M, Gudex C, Lloyd A, Janssen M, Kind P, Parkin D, et al. Development and preliminary testing of the new five-level version of EQ-5D (EQ-5D-5L). Qual Life Res. 2011;20(10):1727–36. 10.1007/s11136-011-9903-x 21479777 PMC3220807

[22] Versteegh MM, Vermeulen KM, Evers SMAA, de Wit GA, Prenger R, Stolk EA. Dutch tariff for the five-level version of EQ-5D. Value Health. 2016;19(4):343–52. 10.1016/j.jval.2016.01.003 27325326

[23] Fayers P, Aaronson NK, Bjordal K, Groenvold M, Curran D, Bottomley A. EORTC QLQ-C30 scoring manual. 3rd ed. Brussels: European Organisation for Research and Treatment of Cancer; 2001.

[24] Giesinger JM, Kieffer JM, Fayers PM, Groenvold M, Petersen MA, Scott NW, et al. Replication and validation of higher order models demonstrated that a summary score for the EORTC QLQ-C30 is robust. J Clin Epidemiol. 2016;69:79–88. 10.1016/j.jclinepi.2015.08.007 26327487

[25] Trepanier M, Minnella EM, Paradis T, Awasthi R, Kaneva P, Schwartzman K, et al. Improved disease-free survival after prehabilitation for colorectal cancer surgery. Ann Surg. 2019;270(3):493–501. 10.1097/SLA.0000000000003465 31318793

[26] van der Hulst HC, van der Bol JM, Bastiaannet E, Portielje JEA, Dekker JWT. The effect of prehabilitation on long-term survival and hospital admissions in older patients undergoing elective colorectal cancer surgery. Eur J Surg Oncol. 2024;50(4):108244. 10.1016/j.ejso.2024.108244 38452716

[27] Breedveld-Peters JJL, Koole JL, Muller-Schulte E, van der Linden BWA, Windhausen C, Bours MJL, et al. Colorectal cancers survivors’ adherence to lifestyle recommendations and cross-sectional associations with health-related quality of life. Br J Nutr. 2018;120(2):188–97. 10.1017/S0007114518000661 29658446

[28] Ferreira V, Agnihotram RV, Bergdahl A, van Rooijen SJ, Awasthi R, Carli F, et al. Maximizing patient adherence to prehabilitation: what do the patients say? Support Care Cancer. 2018;26(8):2717–23. 10.1007/s00520-018-4109-1 29478189

[29] Huang HC, Szwerinski NK, Nasrallah C, Huang Q, Chopra V, Venditti EM, et al. Lifestyle change program engagement in real-world clinical practice: a mixed-methods analysis. Transl Behav Med. 2023;13(3):168–82. 10.1093/tbm/ibac098 36694916 PMC10068905

[30] Livia B, Elisa R, Claudia R, Roberto P, Cristina A, Emilia ST, et al. Stage of change and motivation to a healthier lifestyle before and after an intensive lifestyle intervention. J Obes. 2016;2016:6421265. 10.1155/2016/6421265 27239339 PMC4864539

[31] Boyle H, Fullbrook A, Wills A, Veal I, Peat N, Al-Noor Z, et al. Multimodal prehabilitation service for patients with colorectal cancer: the challenges of implementation. BMJ Open Qual. 2023;12(2):e002064. 10.1136/bmjoq-2022-002064 PMC10230997 37220992

[32] Powell R, Davies A, Rowlinson-Groves K, French DP, Moore J, Merchant Z. Acceptability of prehabilitation for cancer surgery: a multi-perspective qualitative investigation of patient and ‘clinician’ experiences. BMC Cancer. 2023;23(1):744. 10.1186/s12885-023-10986-0 37568097 PMC10416438

[33] Wang R, Yao C, Hung SH, Meyers L, Sutherland JM, Karimuddin A, et al. Preparing for colorectal surgery: a qualitative study of experiences and preferences of patients in Western Canada. BMC Health Serv Res. 2022;22(1):730. 10.1186/s12913-022-08130-y 35650598 PMC9161453

[34] Tsimopoulou I, Pasquali S, Howard R, Desai A, Gourevitch D, Tolosa I, et al. Psychological prehabilitation before cancer surgery: a systematic review. Ann Surg Oncol. 2015;22(13):4117–23. 10.1245/s10434-015-4550-z 25869228

