# Supplementary material for: Long-term lifestyle effects of prehabilitation in colorectal cancer
Source: Acta Oncol. 2026 May 27;65:45608. doi: 10.2340/1651-226X.2026.45608 (PMC13221714; doi:10.2340/1651-226X.2026.45608)
Supplement: Supplementary file 1 [file AO-65-45608-s1.pdf]

Supplementary material has been published as submitted. It has not been copyedited, or typeset by Acta Oncologica

Supplementary Table 1. Physical activity outcomes per time-point per group (unadjusted p-values).

	T6			T12			T24		
**SQUASH (median, IQR)**									
**MET hours/week**									
	Prehab n=69	Control n=42	p-value	Prehab n=54	Control n=34	p-value	Prehab n=27	Control n=24	p-value
Sport	16.5 [8.3-28.5]	11.0 [5.8-22.2]	0.380	21.8 [8.6-32.4]	16.5 [7.1-38.4]	0.882	9.3 [4.0-27.8]	13.3 [8.3-39.0]	0.235
General	102.4 [60.3-152.0]	90.1 [52.0-140.4]	0.563	107.6 [55.3-138.4]	87.3 [63.1-141.8]	0.951	77.8 [43.8-170.0]	97.9 [50.9-155.3]	0.604
Total	104.0 [66.8-165.8]	104.4 [59.1-151.1]	0.648	119.1 [69.0-168.2]	105.5 [68.2-149.5]	0.515	104.3 [49.1-179.8]	104.9 [64.1-168.7]	0.713
**Activity score**									
Sport	1200.0 [540.0-2160.0]	780.0 [480.0-1602.5]	0.647	1680.0 [720.0-2688.0]	1200.0 [600.0-3180.0]	0.699	780.0 [345.0-1980.0]	990.0 [675.0-2955.0]	0.224
General	6700.0 [3705.0-10335.0]	6117.5 [3877.5-8985.0]	0.656	6810.0 [3832.5-9515.3]	6240.0 [5010.0-9957.5]	0.913	5430.0 [2460.0-11400.0]	7080.5 [4162.5-9262.5]	0.533
Total	7160.0 [4115.0-11060.0]	6800.0 [4016.3-10095.0]	0.803	7240.0 [5239.5-11752.5]	7317.5 [5207.5-11143.8]	0.659	7290.0 [3360.0-11760.0]	7177.5 [4848.8-12337.5]	0.734

Outcomes are presented as median (IQR). All p-values reported in this table are unadjusted; multiple-comparison corrections are described in the Results section. MET: Metabolic Equivalent of Task.

Supplementary Table 2. Quality of Life outcomes per time-point per group (unadjusted p-values).

	T6			T12			T24		
	Prehab n=75	Control n=43	p-value	Prehab n=59	Control n=36	p-value	Prehab n=30	Control n=25	p-value
**EQ-5D-5L (median, [IQR])**									
EQ-Index	0.91 [0.81-1.00]	0.90 [0.82-1.00]	0.441	0.89 [0.82-1.00]	0.96 [0.85-1.00]	0.287	0.89 [0.77-1.00]	1.00 [0.87-1.00]	0.052
EQ-VAS	83.0 [74.0-91.0]	80.0 [65.0-89.0]	0.174	81.0 [70.0-92.0]	80.5 [70.0-90.0]	0.738	80 [69.8-85.0]	87.0 [72.0-91.5]	0.165
**EORTC QLQ-C30 (median, [IQR])**									
PF	93.3 [80.0-100.0]	93.3 [80.0-100.0]	0.873	93.3 [86.7-100.0]	93.3 [81.7-100.0]	0.333	93.3 [80.0-100.0]	93.9 [86.7-100.0]	0.356
EF	100.0 [75.0-100.0]	100.0 [83.3-100.0]	0.980	91.7 [83.3-100.0]	100.0 [83.3-100.0]	0.259	91.7 [75.0-100.0]	100.0 [89.6-100.0]	0.363
CF	100.0 [83.3-100.0]	100.0 [83.3-100.0]	0.405	100.0 [83.3-100.0]	91.7 [83.3-100.0]	0.264	100.0 [83.3-100.0]	83.3 [79.2-100.0]	0.268
SF	100.0 [83.3-100.0]	100.0 [83.3-100.0]	0.521	100.0 [100.0-100.0]	100.0 [100.0-100.0]	0.863	100.0 [83.3-100.0]	100.0 [95.8-100.0]	0.278
RF	100.0 [66.7-100.0]	100.0 [66.7-100.0]	0.636	100.0 [83.3-100.0]	100.0 [83.3-100.0]	0.508	100.0 [66.7-100.0]	100.0 [95.8-100.0]	0.049
GHS	83.3 [75.0-100.0]	83.3 [66.7-83.3]	0.133	83.3 [66.7-100.0]	83.3 [70.8-100.0]	0.170	83.8 [66.7-91.7]	83.3 [75.0-100.0]	0.181
SUM	92.2 [84.7-97.4]	94.6 [85.7-96.4]	0.960	94.2 [87.5-99.0]	93.3 [87.1-99.0]	0.593	89.2 [82.1-97.1]	93.7 [87.3-98.1]	0.191

Outcomes are presented as median [IQR]. All p-values reported in this table are unadjusted; multiple-comparison corrections are described in the Results section. PF: Physical functioning, EF: Emotional functioning, CF: Cognitive functioning, SF: Social functioning, RF: Role functioning, GHS: Global health score, SUM: Summary score.
